# CCR2 and coronary artery disease: a woscops substudy

**DOI:** 10.1186/1756-0500-3-31

**Published:** 2010-02-02

**Authors:** David J Dow, Alex D McMahon, Ian C Gray, Chris J Packard, Pieter HE Groot

**Affiliations:** 1 GlaxoSmithKline Medicines Research Centre, Gunnels Wood Road, Stevenage, SG1 2NY, UK; 2 Robertson Centre for Biostatistics, Boyd Orr Building, University of Glasgow, G12 8QQ, UK; 3 Division of Medical Sciences, University of Glasgow, Western Infirmary, Glasgow, G11 6NT, UK; 4 Current Address: Takeda Singapore, 10 Biopolis Road, 03-01/02 Chromas, 138670, Singapore

## Abstract

**Background:**

Several lines of evidence support a role for CCL2 (monocyte chemotactic protein-1) and its receptor CCR2 in the development of atherosclerosis. The aim of the present study was to determine the association of the CCR2 Val64Ile polymorphism with the development of coronary artery disease in the WOSCOPS study sample set.

**Findings:**

A total of 443 cases and 1003 controls from the West of Scotland Coronary Prevention Study (WOSCOPS) were genotyped for the Val64Ile polymorphism in the CCR2 gene. Genotype frequencies were compared between cases and controls. The CCR2 Val64Ile polymorphism was found not to be associated with coronary events in this study population (odds ratio 1.15, 95% CI 0.82-1.61, p = 0.41).

**Conclusions:**

This case-control study does not support an association of the CCR2 Val64Ile polymorphism with coronary artery disease in the WOSCOPS sample set and does not confirm a possible protective role for CCR2 Val64Ile in the development of coronary artery disease.

## Introduction

There is increasing evidence from numerous studies to indicate that the over-recruitment of leukocytes, especially monocytes, is central to the pathology of atherosclerosis [1-4]. Consequently, atherosclerosis is considered now to be an inflammatory disease. Chemokines are inflammatory chemotactic cytokines that play a role in leucocyte recruitment and the chemokine CCL2 (monocyte chemoattractant protein-1 or MCP-1) has been found to be highly expressed in human atherosclerotic plaques [5]. CCL2 attracts monocytes as well as T lymphocytes and mediates its effects by binding to G protein coupled receptors expressed on the surface of target cells [6]. The most prominent receptor for CCL2 is CCR2 [7] and its binding to this receptor has been shown to result in recruitment of monocytes into the subendothelial space [8]. Further evidence for the role of CCL2 and its receptor CCR2 in the pathology of atherosclerosis has come from gene knock-out experiments in mice. CCR2 (-/-) mice show defects in leukocyte adhesion and monocyte/macrophage recruitment [9]. The absence of CCR2 on macrophages protects those mice from developing atherosclerosis [10-12]. Moreover, mice lacking the ligand CCL2 (-/-) also showed decreased lesion size and a significant reduction of macrophages in lesions [13]. In addition, overexpression of CCL2 results in an increase in macrophage number within the lesions [14]. Taken together, these studies strongly suggest a direct role for CCL2 and CCR2 in monocyte recruitment and in the pathology of atherosclerosis. The CCR2 gene comprises two exons and is subject to alternative splicing [6]. A G to A coding polymorphism in exon 2 of the gene has been reported which results in a Val to Ile substitution at position 64. Evidence for this polymorphism having an effect on the function of CCR2 comes from studies of HIV infected individuals. CCR2 and CCR5 are required for the entry of the virus into lymphocytes. Those individuals carrying the CCR2 Ile allele were found to progress to AIDS 2-4 years later than individuals homozygous for the common Val allele [15]. We and others have reported on the role of this polymorphism in the development of coronary artery disease, but the findings do not all appear to be in agreement. In our previous study [16] we looked at the effect of the Val64Ile polymorphism in a cohort of first degree relatives of patients with premature coronary artery disease. The extent of coronary artery calcification was found to be significantly lower in subjects with the CCR2 Ile64 variant than in subjects carrying two Val64 alleles which we interpreted as being beneficial. A similar conclusion was drawn by Szalai *et. al*. [17] based on the absence of any CCR2 Ile64 homozygotes in the cases group of a CAD case-control study. In contrast, Ortlepp *et. al*. [18] found the Ile64 allele to be associated with an increased risk for MI (myocardial infarction) and heart failure in subjects of age <65 y in a large cohort of patients who underwent left ventricular catheterization and also Gonzalez *et. al*. [19] were unable to find a protective effect of the Ile64 allele MI in a case control study. To investigate further our previous suggestion for a protective effect of Ile64 in coronary atherosclerosis, we examined the role of the Val64Ile polymorphism in a nested case-control sample from the West of Scotland Coronary Prevention (WOSCOPS) study.

## Methods

### Woscops

The West of Scotland Coronary prevention study (WOSCOPS) was a randomised, double-blind, placebo controlled trial of pravastatin [20]. The aim of the study was to examine the effect of pravastatin in preventing non fatal MI or death from coronary artery disease. A total of 6595 men aged 45-64 years (mean 55.2 years) with raised cholesterol levels were randomised in equal numbers to pravastatin after initial screening of approximately 81,000 subjects. Individuals had no history of myocardial infarction and had normal renal and hepatic function. Cases were defined as those individuals who, during the course of the five year study, died from definite coronary artery disease (CAD) or who experienced non-fatal acute myocardial infarction, or who were subject to re-vascularisation. Controls, also taken from the original cohort of 6595 men with raised cholesterol levels, did not have a cardiac event. Each case was matched with 2 controls on the basis of age and smoking status. In this present study 443 cases and 1003 controls were examined where specimens had been stored for genetic analysis. 182 of the cases (41%) and 509 controls (51%) received pravastatin treatment. Ethical approval for the study was received from each of the four ethical committees in the west of Scotland (Lanarkshire, Glasgow, Argyle & Clyde and Dumfries & Galloway). Informed consent for the present genetics study was received from all individuals.

### Val64Ile Genotyping

The samples in this study were genotyped using the amplifluor genotyping method [21]. Using this method, two allele specific primers are designed, each of which is complementary to either the wild type or variant allele of the polymorphism at the 3' most base of the primer. Each primer has a 5' tail which enables it to bind to one of two "uniprimers". A "common" primer is the reverse primer for the PCR reaction. Each uniprimer is labelled with a fluorescent dye (JOE or FAM, Applied Biosystems). During the course of the reaction, uniprimers fluoresce if the SNP (Single Nucleotide Polymorphism) allele is complementary to the end base of the primer. The Val64Ile polymorphism is a G to A polymorphism situated in exon 2 of the b isoform of CCR2. The presence of a high fluorescence signal for FAM indicates a G/G genotype, a high level of JOE fluorescence indicates an A/A genotype. Intermediate levels of FAM and JOE fluorescence indicate a heterozygous G/A genotype. The fluorescence measurements were carried out using an ABI 7700 sequence detection system (Applied Biosystems).

The primer sequences used were as follows:

Allele 1 G **GAAGGTGACCAAGTTCATGC**TTTTGCAGTTTATTAAGATGAGGAC

Allele 2 A **GAAGGTCGGAGTCAACGGAT**TTTTGCAGTTTATTAAGATGAGGAT

Common 1 GCTCTACTCGCTGGTGTTCAT

The PCR product size was 74 bp.

### Statistical analysis

The WOSCOPS Biobank is a generic tool designed for various case-control studies. The power of the design depends on the gene frequency of the genotype being studied. A case-control study with 443 cases and 1003 controls can detect with 80% power a rate-ratio of 1.71 for a gene frequency of 5%, a rate-ratio of 1.50 for a gene frequency of 10%, and a rate-ratio of 1.42 for a gene frequency of 15%. A chi-squared analysis was used to determine if the distribution of genotypes was in Hardy-Weinberg equilibrium. Conditional logistic regression was used to assess the effect of individual alleles by calculating odds ratios with associated 95% confidence interval.

## Results

### Study subjects

The CCR2 Val64Ile genotype was determined in a total of 443 cases and 1003 control individuals from the WOSCOPS sample set. The CCR2 polymorphism was found to be in Hardy-Weinberg equilibrium (Cases: chi-squared = 2.25, p = 0.13; Controls: chi-squared = 0.02, p = 0.9). Genotype frequencies were as follows: Val/Val 1268 (87.7%), Val/Ile 174 (12%) and Ile/Ile 4 (0.3%). This gave a minor allele frequency for the Ile allele of 0.06. Due to the small number of Ile/Ile homozygotes, we pooled heterozygotes and homozygotes for the minor allele prior to analysis. Table 1 shows the results of statistical analysis. For the clinical endpoint of death from definite coronary artery disease (CAD), non-fatal acute myocardial infarction, or a re-vascularisation event, the odds ratio for the Ile allele was 1.15 (95% CI 0.82 - 1.61).

**Table 1:** CCR2 Val64Ile genotypes and allele frequencies for cases and controls.

Phenotype and Genotype	Cases (n = 443) (frequency %)	Controls (n = 1003) (frequency %)	Odds Ratio (Ile/Ile or Val/Ile)	95% confidence interval	P-value
Death/Non-fatal event					
					
Val/Val	384 (86.7)	884 (88.1)			
Val/Ile or Ile/Ile (pooled)	59 (13.3)	119 (11.9)	1.15	0.82 - 1.61	0.410
					
Val	0.93	0.94			
Ile	0.07	0.06			

In addition, the analysis of the re-vascularisation end point only produced an odds ratio of 0.78 (95% CI 0.33 - 1.87), the odds ratio for the non-fatal acute myocardial infarction endpoint was 1.28 (95% CI 0.89 - 1.86) and for the death from definite coronary artery disease (CAD) endpoint it was 0.96 (95% CI 0.45 - 2.08). All three sub-group analyses were non-significant.

## Discussion

For a complex pathology such as coronary artery disease, there are likely to be many susceptibility genes which play a role in determining the phenotype [22]. Based upon previous work implicating the CCR2 gene in the progression of atherosclerosis, we sought to further assess the role of the Val64Ile polymorphism in the clinical endpoint of coronary artery disease. In this study we examined the genotypes of 443 cases and 1003 control individuals from the WOSCOPS study. The results obtained for both the combined and single endpoint analyses did not indicate a significant difference in genotype frequencies between cases and controls and so do not suggest a significant role for this polymorphism in the development of coronary artery disease. A number of recent studies have looked at the association of the CCR2 Val64Ile polymorphism and clinical endpoints associated with atherosclerosis. In our previous study the extent of coronary artery calcification was significantly lower in subjects with the CCR2 Ile64 variant than in subjects carrying two Val64 alleles [16] suggesting a beneficial effect for the Ile64 allele. Coronary artery calcification has previously been shown to be correlated with the extent of coronary atherosclerotic lesions [23]. The results of our present study are not in agreement with our previous study and there are several potential explanations for this. Firstly the definition of cases was different. Valdes *et al*. defined cases as male or female first degree relatives of subjects with premature coronary artery disease and there were various exclusion criteria including smoking status. Subjects were non-Hispanic whites from the Pennsylvania area. In the Woscops study, all subjects were men from the west of Scotland with pre-determined cut off values for cholesterol and LDL/cholesterol and smokers were included. The phenotypes are also different between the studies. Valdes *et al*. measured coronary artery calcification (CAC) using tomography. In Woscops the endpoints were death from coronary artery disease (CAD), non-fatal acute myocardial infarction, or re-vascularisation. In addition the sample sets were different with 400 cases and 1003 controls for this study and 662 subjects in the CAC study. In Valdes *et al*, the genotype frequencies were compared between individuals in a high or low CAC value group whereas the cases in Woscops were those with the aforementioned endpoints. Also in Woscops, each case was matched to 2 controls. It is possible that these differences in study design or geographical location of subjects could account for the different conclusions from the two studies examining the same Val64 Ile polymorphism. Other evidence attributing a role for CCR2 in the development of atherosclerosis has been shown [18]. In contrast, here the Ile allele was found to be positively associated with myocardial infarction and heart failure in patients less than 65 years of age. There was no association of the CCR2 genotype with atherosclerosis, but the CCR2 Ile allele appeared to predispose patients to myocardial infarction before 65 years of age. In other work, the role of CCR2 Val64Ile was not supported in studies of both myocardial infarction and coronary artery disease as diagnosed by angiography in patients versus controls [19,24]. Also recently Bjarnadottir *et al*. [25] were unable to find any effect of the CCR2 Val64Ile mutation in a large Icelandic retrospective nested case-control study of MI survivors. Again it cannot be excluded that the different conclusions of these studies could be related to differences in study design, clinical endpoints of the coronary artery disease phenotype or that subjects were derived from different geographical populations where there are differences in genetic or environmental factors [26]. The WOSCOPS sample set is a large, well characterised cohort comprising individuals from the same geographical location. In summary, the results of this present study do not support a role for the CCR2Val64Ile polymorphism in the endpoints of death from coronary artery disease (CAD), non-fatal acute myocardial infarction, or re-vascularisation.

## Conclusions

This CCR2 association study does not confirm a role for the CCR2 Val64Ile polymorphism in the development of coronary artery disease.

## Competing interests

DJD is an employee of GlaxoSmithKline. ICG and PHEG are former employees of GlaxoSmithKline.

## Authors' contributions

DJD - Designed, planned and managed the CCR2 study, designed genotyping assays and wrote the manuscript. ADM - Designed the CCR2 study, preformed statistical analysis of the genotyping data and edited the manuscript. ICG - Designed the CCR2 study and edited the manuscript. CJP - Advised on the study, is a main investigator of the WOSCOPS study and edited the manuscript. PHEG - managed the study and edited the manuscript. All authors read and approved the final manuscript.
